# Calcium- and Sodium-Rich Food Intake among Koreans with and without Metabolic Syndrome: Cross-Sectional Analysis of the Korean Genome and Epidemiology Study

**DOI:** 10.3390/nu16152439

**Published:** 2024-07-26

**Authors:** Byeonggeun Choi, Jiyoon Kim, Yeonjin Kim, Jiae Shin, Sang-Ah Lee

**Affiliations:** 1 Interdisciplinary Graduate Program in Medical Bigdata Convergence, Kangwon National University School of Medicine, Chuncheon 24341, Republic of Korea; kyo910814@kangwon.ac.kr (B.C.); bbkeye1229@naver.com (Y.K.); jiaejshin@gmail.com (J.S.); 2 Department of Preventive Medicine, Kangwon National University School of Medicine, Chuncheon 24341, Republic of Korea; jiyoonk526@gmail.com

**Keywords:** metabolic syndrome, dietary calcium, dietary sodium, Republic of Korea

## Abstract

Background: The prevalence of metabolic syndrome (MetS) is increasing. While calcium and sodium are known nutritional factors used for managing MetS, few studies have focused on food-based analyses. This cross-sectional study examined the distribution of calcium- and sodium-rich food intake among Koreans with MetS. Methods: This cross-sectional analysis evaluated 130,423 participants from the Health Examinees-Gem cohort study. Foods contributing up to 90% of the calcium and sodium intake were selected from the semi-quantitative food frequency questionnaire, and consumption levels were calculated. MetS was defined as satisfying three or more criteria from the National Cholesterol Education Program Adult Treatment Panel III. The results are presented as odds ratios (OR) with an interquartile range (OR_IQR_) and 95% confidence interval. Results: Participants with MetS showed a low calcium intake (OR_IQR_ = 0.95 and 0.92 for men and women, respectively), low consumption of dairy products (OR_IQR_ = 0.92 and 0.89), beverages except for coffee or green tea (OR_IQR_ = 0.97 and 0.96), and bread (OR_IQR_ = 0.96 and 0.94). Men with MetS consumed high total sodium (OR_IQR_ = 1.04), and large amounts of Kimchi (OR_IQR_ = 1.03), fermented paste (OR_IQR_ = 1.04), and noodles (OR_IQR_ = 1.07). Women with MetS consumed more Kimchi than those without MetS (OR_IQR_ = 1.04). The odds ratio for the low calcium and high sodium group compared to the high calcium and low sodium group was 1.26. Conclusion: The MetS group consumed less calcium-rich foods and more sodium-rich foods than those without MetS. Patients with MetS might benefit from precise recommendations of high calcium-rich and low sodium-rich foods.

## 1. Introduction

Metabolic syndrome (MetS) is characterized by a combination of metabolic traits, including abdominal obesity, hyperglycemia, hypertension, and dyslipidemia [1]. These factors contribute to diabetes mellitus or cardiovascular disease (CVD). The global prevalence of MetS has risen to 31.4% [2] and continues to increase. The prevalence of MetS in Korea also increased from 21.6% to 22.9% between 2007 and 2018, according to the Korean National Health and Nutrition Examination Surveys (KNHANESs) [3].

Dietary changes are one of the main therapeutic strategies for MetS. Understanding the protective effect of macrominerals in food is important for managing MetS. Calcium and sodium are involved in fluid balance, membrane potential, hormone secretion, and muscular contractions in the body [4,5]. Calcium consumption is related to the serum lipid profile and lipolysis, and poor dietary calcium induces calcitriol secretion, elevating blood pressure [6,7]. High sodium consumption is associated with hypertension [8,9].

A previous study reported that dietary calcium consumption is a protective factor in MetS [10]. However, other studies found an insignificant association between dietary calcium and MetS [11]. Furthermore, a food-based approach is promising for the general population because we consume food rather than nutrients. Prior studies have analyzed the association between dairy products, a well-known calcium-rich food, and MetS [7,12]. However, few studies have been conducted on the association between calcium-source foods, other than dairy products, and MetS. Koreans consume lower amounts of calcium and dairy products than the Western population [13], implying the necessity to analyze the potential impact of calcium-rich Korean foods on MetS.

A reduction in dietary sodium might lower blood pressure and body fat and improve insulin resistance [14,15]. However, sodium sources and consumption patterns differ by race, gender, region, and nation. For example, on average, Koreans consume 19.6% of their sodium from Kimchi [16]. The mean sodium intake in Korea (5.0 g/day) was ranked second among countries in the world [17].

Much research has been conducted on the dietary patterns and the prevalence/incidence of metabolic syndrome in Korean [18,19,20]. In particular, the effect of CA and Na intake on the development of metabolic syndrome are well established [21,22,23,24,25]. Nevertheless, a food-based approach is useful in presenting guidelines for the prevention of metabolic syndrome. Therefore, this study aimed to identify the distribution of calcium- or sodium-rich food intake among Koreans with MetS and the effect of calcium and sodium intake on MetS using the Health Examinees-Gem (HEXA-G) cohort.

## 2. Materials and Methods

### 2.1. Study Population

HEXA-G is a large-scale cohort study carried out with the community-based Korean Genome Epidemiology Study (KoGES) to determine the association between dietary and chronic diseases. The HEXA-G study recruited 141,971 participants from 38 hospitals and medical examination centers in the Republic of Korea from 2004 to 2013. Informed consent forms were obtained from all participants. The questionnaires, including demographic and lifestyle information, were collected by trained interviewers. Detailed information on the HEXA-G study design has been previously described [26].

**Figure 1:**
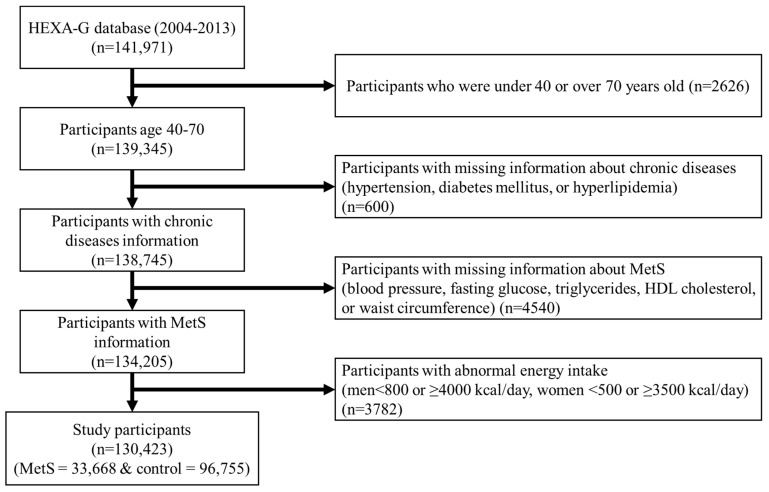
Selection of study participants. HEXA-G, Health Examinees-Gem; MetS, metabolic syndrome; and HDL, high-density lipoprotein.

The selection criteria for this study were as follows: we excluded (1) 2626 participants under 40 or over 70 years old; (2) 600 participants without information about chronic diseases such as hypertension, diabetes mellitus, or hyperlipidemia history; (3) 4540 participants with no information on MetS measurement factors such as blood pressure, fasting glucose, triglycerides (TG), high-density lipoprotein (HDL) cholesterol, or waist circumference (WC); and (4) 3782 participants with implausible energy intakes (<800 or ≥4000 kcal/day for men; <500 or ≥3500 kcal/day for women). As a result, 130,423 subjects (43,850 men and 86,573 women) were included in this study (Figure 1).

### 2.2. Dietary Consumption Assessment

Dietary consumption was assessed using the semi-quantitative food frequency questionnaire (SQ-FFQ), which includes 106 food items developed for the KoGES [27,28,29]. The frequency of food consumption was divided into nine levels (from “never” to “three times or more a day”), while portion size was divided into three levels (one-half, one, and one-and-a-half portions). The validity and reproducibility of the FFQ have been verified [30]. The FFQ and the 12-day diet records had adjusted correlation coefficients of 0.23 to 0.64 for validity (Ca 0.41). The reproducibility of two FFQs one year apart averaged 0.45 for all nutrient intakes (Ca 0.54) and 0.39 for nutrient densities (Ca 0.51). To examine calcium and sodium consumption in the food base, foods were selected based on calcium and sodium intake contributions of up to 90%. The dietary calcium and sodium consumption was calculated by multiplying the daily food consumption frequency by the calcium and sodium contents of the foods.

### 2.3. Definition of Metabolic Syndrome

MetS was defined according to the criteria of the National Cholesterol Education Program Adult Treatment Panel III (NCEP-ATP III) [31] with modifying WC criteria for Asians [32]. Subjects with more than three of the following conditions were diagnosed with MetS: WC ≥ 90 cm for men and ≥80 cm for women, TG ≥ 150 mg/dL or using medication for the treatment of high TG, HDL cholesterol ≤ 40 mg/dL for men and ≤50 mg/dL for women, systolic blood pressure (SBP) ≥ 130 mmHg, diastolic blood pressure (DBP) ≥ 85 mmHg or using medication for the treatment of high blood pressure, fasting blood glucose ≥ 100 mg/dL or using medication for the treatment of high fasting blood glucose.

### 2.4. Covariation Variables

Demographic and lifestyle characteristics included age, marital status, education, employment, family income, smoking, drinking, exercise, body mass index (BMI), and total energy intake from the questionnaire. Patients were divided into three age groups: 40–49, 50–59, and 60–69 years old. Marital status (yes, no), education (≤12, >12 years), family income (<USD 3000, ≥USD 3000/month), and employment (occupied, unoccupied) were divided into two categories. BMI was calculated by dividing the weight in kilograms by the height in meters squared (kg/m^2^). Smoking status was divided into three categories: non-smokers (≤400 cigarettes in their lifetime), former smokers (≥400 cigarettes in their lifetime before the start of the KoGES), and current smokers (≥400 cigarettes at the baseline). Drinking statutes were categorized into two groups: non-current drinkers (no alcohol consumption at baseline) and current drinkers (consuming alcohol at baseline). Regular exercise was defined as participation in physical activities that cause sweating (at least 5 days a week, lasting at least 30 min per session).

### 2.5. Statistical Analysis

To present the distribution of demography and lifestyle according to exposure factors, continuous variables were presented using the median (Q1–Q3), and categorical variables were presented using proportions (%); age, BMI, and total energy intake were continuous, but the other exposure factors were categorical.

To determine the list of calcium- and sodium-rich foods, a linear regression model was used to select foods that contributed 90% of total calcium and sodium intake and foods that could explain up to 90% of the inter-individual variation the semi-quantitative food frequency questionnaire [33].

The effects of total calcium and sodium intake, including calcium and sodium-rich food, on MetS were assessed using multivariable logistic regression models according to interquartile range (IQR) after adjustment for demographic and lifestyle characteristics: age, marital status, education, employment, income, smoking, drinking, exercise, BMI, and total energy intake. To examine the potential synergistic effect of calcium and sodium on MetS, the groups were divided into low- and high-intake groups based on the median intake value. Statistical significance was defined as a *p*-value < 0.05 (2-tailed test). SAS (version 9.4; SAS Institute Inc., Cary, NC, USA) was used to analyze all data.

## 3. Results

It was found that there was no significant difference according to the level of exposure factors (Table 1), although we observed a small difference according to the the level of exposure. The differences in demographic and lifestyle factors according to exposure factors are as follows: Compared to participants in the lowest quartile (Q1), those in the highest quartile (Q4) of calcium intake tended to have graduated from high school, have a higher family income, exercise regularly, and consume more total energy. The highest sodium consumers (Q4) were mostly occupied, were current drinkers, exercised regularly, and had a higher energy intake on average compared to participants with the lowest sodium intake. There were no significant differences in the average age and BMI according to the distribution of calcium and sodium intake for both men and women (Table 1). The socio-demographic and lifestyle characteristics between MetS and control were presented in Table S1.

Table 2 provides the odds ratios according to MetS in terms of total calcium intake and calcium-rich foods contributing 90% of the overall calcium intake. The MetS group exhibited lower calcium intake than the control group in both men (OR_IQR_ = 0.94, 95% CI: 0.91–0.97) and women (OR_IQR_ = 0.92, 95% CI: 0.90–0.94). Both men (OR_IQR_ = 0.92, 95% CI: 0.88–0.95) and women (OR_IQR_ = 0.90, 95% CI: 0.87–0.92) with MetS had a lower dairy product intake than those without MetS. Furthermore, the MetS group demonstrated a decreased consumption of beverages other than coffee or green tea, and this pattern was observed in both men (OR_IQR_ = 0.98, 95% CI: 0.97–0.99) and women (OR_IQR_ = 0.98, 95% CI: 0.97–0.99). Both men (OR_IQR_ = 0.95, 95% CI: 0.94–0.97) and women (OR_IQR_ = 0.94, 95% CI: 0.93–0.95) in the MetS group reported decreased breads consumption. In the case of Kimchi, both men (OR_IQR_ = 1.04, 95% CI: 1.02–1.07) and women (OR_IQR_ = 1.05, 95% CI: 1.03–1.07) with MetS consumed a higher amount of Kimchi in comparison to the control group. Women with MetS had a lower consumption of eggs (OR_IQR_ = 0.95, 95% CI: 0.94–0.96). Men diagnosed with MetS showed an increase in fermented paste intake (OR_IQR_ = 1.04, 95% CI: 1.01–1.06).

The distributions of total sodium and four food items contributing to 90% of total sodium intake are demonstrated in Table 3. Men diagnosed with MetS showed a higher sodium intake compared to the control group (OR_IQR_ = 1.05, 95% CI: 1.02–1.08). In men with MetS, both the consumption of Kimchi (OR_IQR_ = 1.04, 95% CI: 1.02–1.07) and noodles (OR_IQR_ = 1.07, 95% CI: 1.05–1.09) were higher than those in the control group. Women with MetS had a higher intake of Kimchi in comparison to the control group (OR_IQR_ = 1.05, 95% CI: 1.03–1.07) (Table 3).

Table 4 demonstrates the effect of the interaction between sodium and calcium intake stratified by gender and the median value of both calcium and sodium consumption. For men, the median calcium and sodium intakes were 380 and 2486 mg, respectively. The low calcium and high sodium group had an odds ratio of 1.28 (95% CI: 1.17–1.40) for MetS diagnosis compared to the high calcium and low sodium reference group. The median value of calcium consumption in women was 409 mg and that of sodium consumption was 2233 mg. The low calcium and high sodium consumption group exhibited a higher prevalence of MetS in comparison to the high calcium and low sodium group (OR = 1.27, 95% CI: 1.18–1.35). There was no synergistic effect between calcium and sodium intakes in either men or women.

## 4. Discussion

This study analyzed the intake of calcium/sodium-rich food in Koreans with MetS. We identified ten groups of calcium-rich foods and four groups of sodium-rich foods. Participants with MetS consumed calcium-rich foods including dairy products, beverages except for coffee or green tea, and breads less than the control group. Women with MetS showed a lower intake of eggs and calcium-rich food whereas men with MetS consumed fermented paste more than the control group. Both men and women in the MetS group consumed high amounts of kimchi rich in calcium and sodium. Men with MetS had a higher intake of total sodium and noodles than those without MetS. Moreover, no synergistic effect was found between calcium and sodium intake and MetS risk. Participants with low calcium and high sodium intake had a higher prevalence of MetS than those with high calcium and low sodium intake.

The result of low dietary calcium intake among MetS patients is supported by epidemiological studies [10,34]. Calcium’s protective effect against MetS involves reducing the amount of intestinal fat absorption, decreasing intracellular calcium in adipocytes and vascular smooth muscle cells, and modulating the renin–angiotensin system [7,35]. This study showed that the MetS group consumed calcium-rich food and dairy products less than the control group, supporting the results of prospective studies that dairy products were inversely associated with MetS risk [36,37]. Dietary calcium absorption is affected by both the overall calcium content of the food and the presence of components that either facilitate or inhibit calcium absorption. A randomized controlled study reported that a high-dairy diet affected both weight loss and fat loss more than a high-calcium supplement diet in obese participants [6,38]. Thus, the consumption of calcium-rich dairy products is recommended for the management of patients with MetS.

Beverages except for coffee and green tea included carbonated drinks, soybean-based drinks, and other drinks. A cross-sectional study demonstrated that Koreans drinking carbonated soft drinks had low HDL cholesterol levels; however, there was no significant effect on the risk of MetS [39]. A prospective study of the KoGES showed that soy protein from soybean and soy drinks is related to lowering the risk of MetS [40]. In addition, previous papers reported inconsistent results about the effects of coffee and green tea on MetS [41,42]. The high consumption of beverages other than coffee and green tea in the control group may be attributed to the greater protective effect of soybeans against MetS. Considering the potentially stronger protective effect of calcium in soybeans against MetS, individuals with MetS could have benefit of consuming beverages with more calcium than caffeinated drinks or green tea. However, the underlying mechanism is yet to be revealed fully.

A meta-analysis reported that the consumption of eggs rich in calcium was inversely associated with MetS [43]. Bread consumption, which accounted for calcium intake, was inversely associated with serum triglycerides and MetS in men [44]. A randomized crossover study among women revealed that calcium absorption through leavened whole-wheat bread was enhanced compared to milk or co-ingested milk [45]. Our result suggested that calcium consumption through eggs and bread may alleviate MetS, particularly in women with a lower calcium intake than in men [46].

A meta-analysis concluded that MetS patients have higher sodium levels than healthy individuals, as supported by our results [47]. Several studies in the Korean population found that a high sodium intake increased the risk of MetS [21,22,48]. While they estimated sodium intake using a more precise 24 h urinary sodium excretion method, our findings align with these previous outcomes. A sodium-rich diet is associated with MetS components, such as dyslipidemia, hypertension, type 2 diabetes mellitus, and insulin resistance [49].

The MetS group showed a high consumption of Kimchi; however, fermented paste and noodles consumption was high in men with MetS, but not in women counterpart. A prospective study in Korean adults observed that a high intake of vegetable calcium such as Kimchi is inversely associated with MetS components, which is inconsistent with our result [24]. On the other hand, a cross-sectional study of KNHANES reported that Kimchi intake was not associated with MetS in men [24].

Although the absorption rate of Kimchi is lower compared to typical calcium-rich foods such as milk [50], the calcium absorption rate from Kimchi could not have differed between the case and control group, considering the non-differential misclassification effect [51], which could suggest an underestimation of the association between MetS and calcium intake from Kimchi. Kimchi and fermented paste were calcium-rich foods, yet they required a large amount of salt to promote a salty taste. Thus, despite the significant contribution of calcium intake through Kimchi, the relatively high amount of sodium in Kimchi might offset the protective effect of calcium intake on MetS. The high sodium content of fermented paste might similarly contribute to an increased risk of MetS in men. Moreover, Kimchi and fermented paste are often consumed alongside white rice which is rich in carbohydrates, and the highest sodium intake group (Q4) also exhibited the highest total energy intake, which was associated with MetS components [52].

Noodles are composed of flour, starch, salt, and salt substitutes. According to a randomization study using cohorts in KoGES, the high noodle consumption group showed higher sodium intake, and high noodle intake was positively associated with the risk of MetS, which is consistent with our results [53]. A potential explanation for the gender differences in study outcomes may be related to the higher consumption level of sodium-rich food in men. In this study population, a higher percentage of men were current smokers and drinkers than women; this lifestyle pattern is associated with excessive sodium consumption, as supported by A KNHANES study [54]. However, even after adjusting for these confounding factors, men with MetS consumed more Kimchi, fermented paste, and noodles than healthy men. Women may be influenced by the estrogen-mediated suppression of an increased sodium appetite [55]. There might be a contribution of gender-specific criteria for abdominal obesity and MetS components and discrepancies in reporting food consumption between men and women.

The present study had several limitations. Firstly, the cross-sectional design of this study could not demonstrate a causal relationship between calcium/sodium-rich food intakes and MetS. Secondly, self-reporting via the SQ-FFQ method tends to overestimate or underestimate the amount of calcium/sodium intake. If non-differential misclassification is adjusted, the results could show a stronger association between calcium/sodium-rich foods and the MetS group [51]. Thirdly, patients with diabetes mellitus, hypertension, or dyslipidemia might have changed their diet after diagnosis. Although our findings did not reflect the present consumption and longitudinal dietary variations, they constituted 0.84 percent of this cohort. Fourth, there was a lack of available data about individual medication histories that could potentially affect the excretion of calcium and sodium [56].

Despite these limitations, the current study had several strengths. First, this study analyzed a large population of Korean adults obtained from the HEXA-G database. Second, the validity of the FFQ used in this study was established in this population [30]. Furthermore, we approached food-based calcium and sodium intake and evaluated food consumption patterns among MetS patients. This study analyzed commonly consumed Korean foods based on a calcium/sodium intake that is distinctly different from Western diets.

## 5. Conclusions

Koreans with MetS consume less calcium and more sodium. MetS patients showed low consumption of dairy products, beverages except for coffee and green tea, and breads, whereas the intake of Kimchi was higher than that of the healthy control group. The results of this study provide a basis for making dietary recommendations for the management of MetS. Further cohort studies and randomized controlled trials are needed to establish a causal relationship between calcium- or sodium-rich food consumption and MetS.

## Figures and Tables

**Table 1:** Distribution of socioeconomic and lifestyle factors of participants according to quartiles of calcium and sodium intake.

	Men (n = 43,850)				Women (n = 86,573)			
	Q1	Q2	Q3	Q4	Q1	Q2	Q3	Q4
**Calcium Intake**								
Age (years, med, Q1, Q3)	54 (47, 61)	54 (47, 60)	53 (46, 60)	53 (46, 60)	52 (47, 61)	52 (47, 60)	52 (46, 60)	52 (46, 60)
Marital status (married, n, %)	10,145 (93.0)	10,288 (94.2)	10,329 (94.5)	10,398 (95.1)	18,229 (84.6)	18,724 (86.8)	18,844 (87.4)	19,042 (88.3)
Education (>12 year, n, %)	3582 (33.2)	3978 (36.7)	4062 (37.6)	4422 (40.8)	3396 (15.9)	4187 (19.6)	4414 (20.6)	4822 (22.6)
Employment (occupied, n, %)	8489 (79.5)	8742 (81.4)	8839 (82.2)	8810 (82.0)	8276 (39.2)	8464 (40.0)	8566 (40.6)	8156 (38.8)
Family income (≥3000 $/month, n, %)	4380 (45.8)	4769 (48.9)	5052 (51.8)	5066 (52.1)	7284 (39.5)	8236 (44.1)	8393 (45.1)	8592 (47.2)
Current smoker *	3486 (31.9)	3545 (32.4)	3516 (32.1)	3398 (31.1)	574 (2.7)	473 (2.2)	486 (2.3)	479 (2.2)
Current drinker *	7815 (71.4)	8115 (74.2)	8011 (73.3)	7948 (72.7)	6318 (29.3)	6745 (31.3)	6674 (30.9)	6680 (31.0)
Regular exercise *	5581 (51.1)	6029 (55.1)	6410 (58.6)	6980 (63.8)	9356 (43.3)	10,647 (49.3)	11,420 (52.9)	12,784 (59.2)
BMI (kg/m^2^, med, Q1, Q3)	24.2 (22.5, 26.0)	24.3 (22.6, 26.1)	24.4 (22.6, 26.2)	24.5 (22.8, 26.2)	23.3 (22.5, 26.0)	23.3 (22.6, 26.1)	23.3 (22.6, 26.2)	23.3 (22.8, 26.2)
Total energy intake (kcal/day, med, Q1, Q3)	1466 (1296, 1644)	1701 (1524, 1,914)	1883 (1676, 2134)	2220 (1928, 2593)	1345 (1296, 1644)	1574 (1524, 1914)	1747 (1676, 2134)	2060 (1928, 2593)
Family history Hypertension *	2624 (23.9)	2646 (24.1)	2724 (24.9)	2657 (24.2)	6659 (30.8)	7006 (32.4)	7150 (33.0)	6890 (31.8)
Family history Diabetes *	1699 (15.5)	1831 (16.7)	1768 (16.1)	1822 (16.6)	4121 (19.0)	4298 (19.9)	4306 (19.9)	4243 (19.6)
Family history Hyperlipidemia *	86 (1.4)	86 (1.4)	91 (1.5)	93 (1.5)	324 (2.7)	380 (3.1)	385 (3.2)	382 (3.3)
**Sodium Intake**								
Age (years, med, Q1, Q3)	54 (48, 61)	54 (47, 61)	53 (47, 60)	52 (46, 60)	52 (48, 61)	52 (47, 61)	52 (47, 60)	51 (46, 60)
Marital status (married, n, %)	10,226 (93.6)	10,272 (94.1)	10,306 (94.3)	10,356 (94.8)	18,046 (83.8)	18,638 (86.5)	18,976 (88.0)	19,179 (89.0)
Education (>12 year, n, %)	4051 (37.4)	4077 (37.6)	3953 (36.5)	3963 (36.7)	3802 (17.8)	4437 (20.7)	4281 (20.0)	4299 (20.2)
Employment (occupied, n, %)	8518 (79.6)	8612 (80.2)	8815 (82.0)	8935 (83.4)	8077 (38.3)	8481 (40.1)	8445 (39.9)	8459 (40.3)
Family income (≥3000 $/month, n, %)	4759 (49.1)	4918 (50.4)	4840 (49.5)	4750 (49.8)	7639 (41.0)	8563 (45.6)	8287 (44.4)	8016 (44.9)
Current smoker *	3089 (28.2)	3417 (31.2)	3586 (32.8)	3853 (35.2)	503 (2.3)	539 (2.5)	464 (2.2)	506 (2.4)
Current drinker *	7580 (69.3)	7976 (72.9)	8112 (74.1)	8221 (75.1)	6206 (28.8)	6742 (31.3)	6618 (30.7)	6851 (31.8)
Regular exercise *	6146 (56.2)	6174 (56.5)	6255 (57.2)	6425 (58.7)	10,801 (50.0)	10,925 (50.6)	11,095 (51.4)	11,386 (52.7)
BMI (kg/m^2^, med, Q1, Q3)	24.2 (22.5, 26.0)	24.3 (22.5, 26.0)	24.3 (22.5, 26.1)	24.6 (22.9, 26.3)	23.2 (22.5, 26.0)	23.2 (22.5, 26.0)	23.4 (22.5, 26.1)	23.5 (22.9, 26.3)
Total energy intake (kcal/day, med, Q1, Q3)	1525 (1326, 1741)	1713 (1485, 1990)	1851 (1623, 2135)	2138 (1839, 2535)	1377 (1326, 1741)	1581 (1485, 1990)	1714 (1623, 2135)	1977 (1839, 2535)
Family history Hypertension *	2692 (24.6)	2703 (24.7)	2741 (25.0)	2515 (22.9)	7033 (32.5)	7039 (32.5)	6888 (31.8)	6745 (31.2)
Family history Diabetes *	1753 (16.0)	1809 (16.5)	1800 (16.4)	1758 (16.0)	4309 (19.9)	4291 (19.8)	4165 (19.2)	4203 (19.4)
Family history Hyperlipidemia *	98 (1.6)	79 (1.2)	95 (1.5)	84 (1.5)	383 (3.1)	393 (3.1)	334 (2.7)	361 (3.4)

* Number of answering “Yes”: n (%).

**Table 2:** Odds ratios with interquartile range of MetS according to total calcium and calcium-contributing foods intake (90% coverage).

	Men (n = 43,850)			Women (n = 86,573)		
	Median Value (Q3–Q1)		OR_IQR_ (95% CI)	Median Value (Q3–Q1)		OR_IQR_ (95% CI)
	MetS (n = 12,640)	Control (n = 31,210)		MetS (n = 21,028)	Control (n = 65,545)	
Calcium (mg)	378 (258)	381 (268)	0.94 (0.91–0.97)	395 (282)	413 (294)	0.92 (0.90–0.94)
Dairy products (g/day)	47 (137.3)	59 (150.5)	0.92 (0.88–0.95)	69 (185.3)	100 (175.0)	0.90 (0.87–0.92)
Vegetables except for Kimchi and Korean-style pickles (g/day)	105 (96.3)	103 (97.6)	0.99 (0.96–1.01)	113 (105.3)	115 (109.2)	1.01 (0.99–1.02)
Kimchi (g/day)	150 (138.1)	150 (129.8)	1.04 (1.02–1.07)	125 (128.0)	112 (119.3)	1.05 (1.03–1.07)
Fishes except for salt-fermented fish (g/day)	34 (35.0)	32 (34.4)	1.00 (0.98–1.02)	30 (34.2)	31 (34.2)	0.99 (0.97–1.01)
Legumes (g/day)	27 (33.1)	26 (33.8)	0.98 (0.96–1.00)	23 (32.5)	26 (34.0)	0.99 (0.98–1.00)
Beverages except for coffee and green tea (g/day)	26 (46.6)	29 (50.1)	0.98 (0.97–0.99)	27 (43.3)	33 (50.2)	0.98 (0.97–0.99)
Seaweeds (g/day)	1.4 (1.73)	1.4 (1.63)	1.01 (0.98–1.04)	1.5 (2.32)	1.5 (2.30)	1.01 (0.99–1.02)
Eggs (g/day)	11 (10.1)	11 (9.6)	0.99 (0.98–1.01)	4 (9.5)	11 (10.4)	0.95 (0.94–0.96)
Fermented pastes (g/day)	4.5 (4.82)	4.3 (4.82)	1.04 (1.01–1.06)	3.9 (4.61)	3.2 (3.39)	1.01 (0.99–1.03)
Breads (g/day)	5.3 (13.58)	6.0 (15.00)	0.95 (0.94–0.97)	4.2 (11.83)	6.0 (17.08)	0.94 (0.93–0.95)

MetS, metabolic syndrome; OR_IQR_, odds ratio with interquartile range after adjusting for age, education, job, income, marital status, drinking state, smoking state, exercise state, body mass index energy intake, family history of hypertension, diabetes, and hyperlipidemia.

**Table 3:** Odds ratios with interquartile range of MetS according to total sodium and sodium-contributing foods intake (90% coverage).

	Men (n = 43,850)			Women (n = 86,573)		
	Median Value (Q3–Q1)		OR_IQR_ (95% CI)	Median Value (Q3–Q1)		OR_IQR_ (95% CI)
	MetS (n = 12,640)	Control (n = 31,210)		MetS (n = 21,028)	Control (n = 65,545)	
Sodium (mg)	2532 (1739)	2467 (1687)	1.05 (1.02–1.08)	2266 (1610)	2224 (1580)	1.03 (1.00–1.05)
Kimchi (g/day)	150 (138.1)	150 (129.8)	1.04 (1.02–1.07)	125 (128.0)	112 (119.3)	1.05 (1.03–1.07)
Fishes except for salt-fermented fish (g/day)	34 (35.0)	32 (34.4)	1.00 (0.98–1.02)	30 (34.2)	31 (34.2)	0.99 (0.97–1.01)
Noodles (g/day)	44 (60.9)	36 (56.6)	1.07 (1.05–1.09)			
Vegetables except for Kimchi and Korean-style pickles (g/day)				113 (105.3)	115 (109.2)	1.01 (0.99–1.02)

MetS, metabolic syndrome; OR_IQR_, odds ratio with interquartile range after adjusting for age, education, job, income, marital status, drinking state, smoking state, exercise state, body mass index, energy intake, family history of hypertension, diabetes, and hyperlipidemia.

**Table 4:** The interaction effects of sodium and calcium on MetS.

		$Na{\;}^{a}$				*p* for Interaction
		MetS/Control (%)		OR (95%CI)		
		Low	High	Low	High	
$Ca{\;}^{b}$	**Men**					0.9563
	High	12.2/13.8	37.3/36.4	Ref.	1.15 (1.06–1.24)	
	Low	36.2/36.8	14.3/13.0	1.14 (1.05–1.23)	1.28 (1.17–1.40)	
	**Women**					0.4853
	High	12.4/14.7	35.0/36.2	Ref.	1.11 (1.05–1.17)	
	Low	36.6/35.6	16.0/13.5	1.18 (1.11–1.25)	1.27 (1.18–1.35)	

^a^ Median of sodium: 2486 mg for men, 2233 mg for women; ^b^ Median of calcium: 380 mg for men, 409 mg for women. The low category is defined as below the median and the high is defined as equal to or more than the median. MetS, metabolic syndrome; OR, odds ratio after adjusting for age, education, job, income, marital status, drinking state, smoking state, exercise state, body mass index, energy intake, family history of hypertension, diabetes, and hyperlipidemia.

## Data Availability

All data and materials used in this study will be available upon reasonable request from the corresponding author.

## Supplementary Materials

The following supporting information can be downloaded at: https://www.mdpi.com/article/10.3390/nu16152439/s1, Table S1. Socio-demographic and lifestyle characteristics according to metabolic syndrome status.
