# Supplementary material for: Calcium- and Sodium-Rich Food Intake among Koreans with and without Metabolic Syndrome: Cross-Sectional Analysis of the Korean Genome and Epidemiology Study
Source: Nutrients. 2024 Jul 26;16(15):2439. doi: 10.3390/nu16152439 (PMC11314467; doi:10.3390/nu16152439)
Supplement: Supplementary file 1 [file nutrients-16-02439-s001.zip › nutrients-3080733-supplementary.pdf]

Table S1. Socio-demographic and lifestyle characteristics according to metabolic syndrome status

	Men (n=43,850)		Women (n=86,573)	
	**Mets (n=12,640)**	**Control (n=31,210)**	**Mets (n=21,028)**	**Control (n=65,545)**
**Age (years, median, Q1, Q3)**	55 (48, 61)	53 (46, 60)	57 (51, 62)	50 (45, 56)
**Marital status (married, n, %)**	11920 (94.3)	29395 (94.2)	17800 (84.7)	57376 (87.5)
**Education (> 12year, n, %)**	4275 (33.8)	11769 (37.7)	2138 (10.2)	14681 (22.4)
**Employment (occupied, n, %)**	9783 (77.4)	25097 (80.4)	6773 (32.2)	26689 (40.7)
**Family income (≥ 3000$/month, n, %)**	5427 (44.9)	13840 (46.4)	5672 (28.1)	26833 (43.2)
**Current smoker (yes, n, %)**	4312 (34.1)	9633 (30.9)	511 (2.4)	1501 (2.3)
**Current drinker (yes, n, %)**	9346 (73.9)	22631 (72.5)	4927 (23.4)	21490 (32.8)
**Regular exercise (yes, n, %)**	6997 (55.4)	18093 (58.0)	10217 (48.6)	34156 (52.1)
**BMI (kg/m², median, Q1, Q3)**	26 (24.5, 27.7)	23.7 (22.1, 25.3)	25.4 (23.7, 27.4)	22.7 (21.2, 24.5)
**Total energy intake (kcal/day, median, Q1, Q3)**	1788.7 (1524.9, 2125)	1781.2 (1516.4, 2116.9)	1617.2 (1351.1, 1923.9)	1652.2 (1358.8, 1976.2)
**Family history Hypertension (yes, n, %)**	3575 (28.3)	7076 (22.7)	7461 (35.5)	20244 (30.9)
**Family history Diabetes (yes, n, %)**	2497 (19.8)	4623 (14.8)	4703 (22.4)	12265 (18.7)
**Family history Hyperlipidemia (yes, n, %)**	110 (1.6)	246 (1.4)	317 (2.7)	1154 (3.2)
